# Correction: Adult mortality trends in Qatar, 1989-2015: National population *versus* migrants

**DOI:** 10.1371/journal.pone.0207104

**Published:** 2018-11-01

**Authors:** The PLOS ONE Staff

## Notice of Republication

This article was republished on October 24, 2018 to include Supporting Information files S1 Table and S2 Table, which were omitted from the originally published article. The publisher apologizes for the error. Please download this article again to view the correct version. The originally published, uncorrected article and the republished, corrected article are provided here for reference.

## Supporting information

S1 File Originally published, uncorrected article. (PDF)

S2 File Republished, corrected article. (PDF)
