# Supplementary figures and images for: Adult mortality trends in Qatar, 1989-2015: National population versus migrants
Source: PLoS One. 2018 Sep 25;13(9):e0203996. doi: 10.1371/journal.pone.0203996 (PMC6155516; doi:10.1371/journal.pone.0203996)

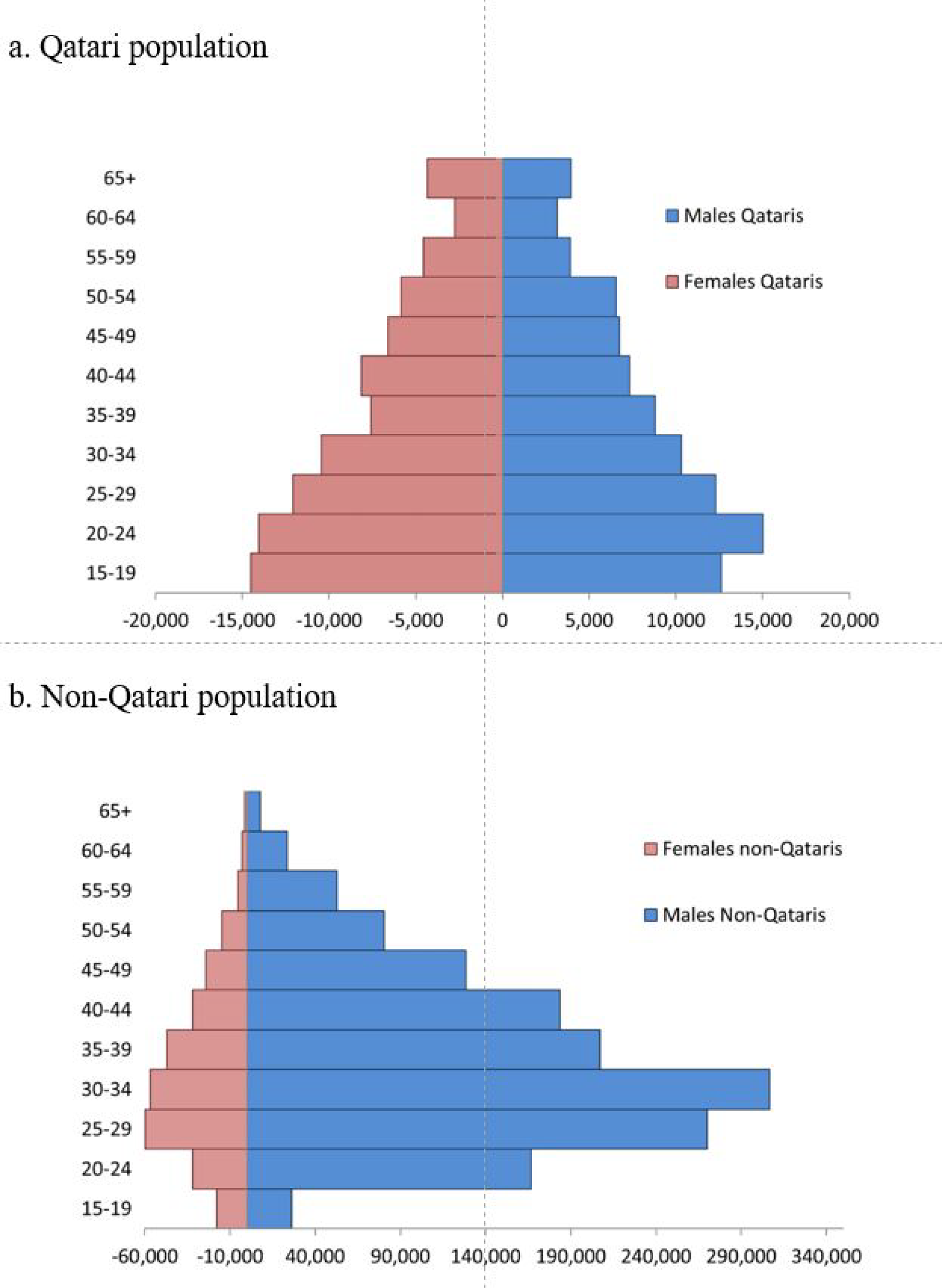

Supplement: S1 Fig — Data source: MDPS’s Census, Population, Housing, and Establishments annual report for the year 2014 [25]. (TIF) [file pone.0203996.s001.tif]
