# Supplementary material for: Correction: Adult mortality trends in Qatar, 1989-2015: National population versus migrants
Source: PLoS One. 2018 Nov 1;13(11):e0207104. doi: 10.1371/journal.pone.0207104 (PMC6211747; doi:10.1371/journal.pone.0207104)
Supplement: S2 File — (PDF) [file pone.0207104.s002.pdf]

PLOS ONE

RESEARCH ARTICLE

Adult mortality trends in Qatar, 1989-2015: National population *versus* migrants

Karima Chaabna*, **Sohaila Cheema, Amit Abraham, Hekmat Alrouh, Ravinder Mamtani**

Institute for Population Health, Weill Cornell Medicine-Qatar, Doha, Qatar

* kac2047@qatar-med.cornell.edu

Abstract

Introduction

With the increase of Qatar's total population, primarily due to the influx of healthy male migrant labor, worldwide attention has been focused on deaths among these migrant workers.

Objective

To describe adult mortality trends in Qataris (nationals) and non-Qataris (migrants) from all causes, cardiovascular and circulatory disease, neoplasms, and injuries, 1989–2015.

Methods

We retrieved Qatar's vital registration data by nationality, sex, age group, year, and codes of the World Health Organization's International Classification of Diseases, Ninth and Tenth Revisions. We assessed age-standardized mortality rate (ASMR) trends in Qatar's total population, in Qataris and non-Qataris using Joinpoint regression.

Findings

During the study period, 26,673 deaths were recorded. In 2015, we estimated 60,716 years of life lost (82% in males) in the overall population. In Qataris (both sexes) and in non-Qatari females, all-cause rate decreased significantly and steadily between 1989–2015. In non-Qatari males, it decreased significantly between 1998–2010 probably attributed to a massive influx of healthy migrants. Yearly rates were significantly lower in non-Qataris over 27 years. Reduction in Qatar's total population rates for all causes and for neoplasms can be partially attributed to the healthy migrant effect. For injuries in males, it was lower in non-Qatari. Remarkably, for falls, cause-specific ASMR in non-Qatari males decreased significantly reaching 2.6/100,000 in 2014, suggesting improved safety in the work environment. However, while young adult males in Qatar die predominantly from injuries, young adult females die from neoplasms.

Conclusion

Our study demonstrates that premature death in young adult males and females in Qatar is predominantly due to injuries and neoplasms respectively. These identified causes of death

Check for updates

OPEN ACCESS

Citation: Chaabna K, Cheema S, Abraham A, Alrouh H, Mamtani R (2018) Adult mortality trends in Qatar, 1989-2015: National population *versus* migrants. PLoS ONE 13(9): e0203996. https://doi.org/10.1371/journal.pone.0203996

Editor: Asharaf Abdul Salam, King Saud University, SAUDI ARABIA

Received: August 1, 2017

Accepted: September 1, 2018

Published: September 25, 2018

Copyright: © 2018 Chaabna et al. This is an open access article distributed under the terms of the Creative Commons Attribution License, which permits unrestricted use, distribution, and reproduction in any medium, provided the original author and source are credited.

Data Availability Statement: Mortality data analyzed in this study are publicly available at MDPS's website in the following link: http://www.mdps.gov.qa/en/statistics1/pages/topicslisting.aspx?parent=Population&child=BirthsDeaths). Population data are also publicly available and obtained from MDPS's website (http://www.qix.gov.qa/portal/page/portal/QIXPOC/Documents/QIX%20Knowledge%20Base/Publication/Labor%20Force%20Researches/labor%20force%20sample%20survey) and UN's website (https://esa.un.org/unpd/wpp/Download/Standard/Population/).

Funding: The authors received no specific funding for this work.

Competing interests: The authors have declared that no competing interests exist.

Abbreviations: AAPC, Average annual percent change; APC, Annual percent change; ASMR, Age-standardized mortality rate; CI, 95% confidence interval; CMF, Comparative Mortality Figure; GBD 2015, Global Burden of Disease Study 2015; ICD-10, International Classification of Diseases, Tenth Revision; ICD-9, International Classification of Diseases, Ninth Revision; MDPS, Ministry of Development Planning and Statistics; *p*-value, Probability-value; UN, United Nations.

are for a large part preventable and should be addressed appropriately to lower premature mortality among young adults in Qatar.

Introduction

The major contributor to Qatar's total population growth has been the influx of healthy male migrant labor [1] to build Qatar's workforce [2, 3]. Consequently, Qatar's total population increased rapidly reaching a population growth rate of 22% in 2005–2010 while it was <4% prior to 2000 [4]; with a resultant change in its age pyramid (S1a and S1b Fig). For 2015, 90% of Qatar's total population aged 15 years and above consisted of non-Qataris [5]. Qatar and other countries of the Gulf Cooperation Council possess unique demographics with high proportions of migrants in their populations, reaching over 80% in the United Arab Emirates [6]. These countries appear to be part of one of the leading labor-importing regions in the world [7] especially from the neighboring Arab and Asian countries [8]. In 2016, 64% of Qatar's total population was from five countries, namely India, Nepal, Bangladesh, Philippines, and Egypt [9]. Recently, worldwide attention has focused on an increased number of deaths among migrant workers within Qatar [10, 11].

Parallel to the demographic challenges, Qatar is grappling with high rates of diabetes and obesity. Qatar is among the top ten countries with the highest diabetes prevalence worldwide [12], at 17% of adult Qataris in 2012 [13]. Additionally, adult obesity in Qatar was 41% in 2012 [13], while the global estimate was 13% [14]. The burden of adult obesity in Qatar is also higher than the other countries of the Gulf Cooperation Council (40% in Kuwait [15], 36% in Bahrain [16], 33% in UAE [17], 30% in Oman [18], and 28% in Saudi Arabia [19]). Qatar has overall benefited from socioeconomic and healthcare system developments [20]. The Global Burden of Disease Study 2015 (GBD 2015) showed decreased all-cause mortality (death from any cause) and cause-specific mortality (death from a specific cause) rates since 1990 in Qatar [21]. Remarkably, these GBD 2015 mortality trends refer to both the Qataris (nationals) and non-Qataris (migrants).

Materials and methods

For assessing the impact of demographic changes, socioeconomic and healthcare system development, and health-based interventions, measuring the magnitude of deaths by population is necessary. Therefore, we evaluated trends over 27 years of all-cause mortality and cause-specific mortality for the three commonest causes of death, namely cardiovascular and circulatory disease, neoplasms, and injuries, in Qataris and non-Qataris [21]. Additionally, we investigated mortality differentials between Qataris and non-Qataris.

In order to estimate mortality rates in Qatar's total population, in Qataris and non-Qataris between 1989–2015, we retrieved vital registration data from the Qatar Vital Statistics Annual Bulletins of the Ministry of Development Planning and Statistics (MDPS). Grouped secondary data, which are publicly available on the Ministry's website [22] were used. Number of deaths by sex, age group (<5, 5–9, 10–19, 20–29, 30–39, 40–49, ≥50), year (from 1989 to 2015), nationality (Qatari/non-Qatari), and codes of the World Health Organization's (WHO) International Classification of Diseases, Ninth and Tenth Revisions (ICD-9 and ICD-10) were retrieved [23, 24]. We retrieved the numbers of all-cause deaths and cause-specific deaths for the three commonest causes of death in Qatar's total population reported by GBD 2015 [21], which are cardiovascular and circulatory disease (ICD-9: 390–459 and ICD-10: I00-I99),

neoplasms (ICD-9: 140–239 and ICD-10: C00-D48), and injuries (ICD-10: V01-Y98 and ICD-9: E811-978).

Publicly available data on Qatar's yearly population size by sex, five-year-age group (from 15 year old to over 75 year old), and nationality were retrieved from MDPS's Census, Population, Housing, and Establishments annual reports for the years 2006–2014 [25]. Annual population growth rates in Qataris were computed by sex and age group for the period 2006–2014. Using these estimated population growth rates, Qatari population size between 1989–2005 and in 2015 was extrapolated assuming constant increase in population between 1989–2015 as observed between 2006–2014. Publicly available data on Qatar's total population size (combining Qataris and non-Qataris) was retrieved by sex and five-year-age group, in 1990, 1995, 2000, 2005, 2010, and 2015 from the World Population Prospect 2015 Revision by United Nations (UN) Population Division [4]. Annual population growth rates were computed by sex and age group for the periods 1990–1995, 1995–2000, 2000–2005, 2005–2010, 2010–2015. To estimate non-Qatari yearly population size by sex, we assumed that sex-specific non-Qatari population growth was similar to sex-specific Qatar's population growth, as the non-Qatari population constituted the vast majority of Qatar's total population– 73% in 1986 to 90% in 2015 [5, 26]. Thus, using these estimated sex-specific population growth rates and sex-specific population size in non-Qataris between 2006–2014, yearly sex-specific non-Qatari population size between 1989–2005 and in 2015 was extrapolated. Computed Qatar's total population size between 1989–2005 and in 2015 was the sum of Qatari and non-Qataris population sizes.

Age-standardized mortality rates (ASMR, weighted average of the age-specific death rates) and corresponding standard errors were estimated using the direct method [27] and the world standard population [28]. Age-standardization method allows comparison of populations with different age-structures [27]. ASMRs were computed for all-causes between 1989–2015. However, recorded number of deaths in 1994, 2008, 2012, and 2013 were not available. Cause-specific age-specific deaths were not available for 2015. Additionally, cause-specific age-specific deaths for cardiovascular and circulatory disease prior to 1991 were also not available. Thus, ASMRs between 1991–2014 for cardiovascular and circulatory disease, hypertensive disease (ICD-9: 401–405 and ICD-10: I10-I14), ischemic heart disease (ICD-9: 410–414 and ICD-10: I20-I25), and cerebrovascular disease (ICD-9: 430–438 and ICD-10: I60-I69) were computed. Cause-specific deaths for neoplasms were not available before 1991, and between 1991–2000 they were reported but grouped for instance as "malignant neoplasms of genitourinary organs". Therefore, we computed ASMRs between 1991–2014 for neoplasms; while, we calculated ASMRs between 2000–2014 for female breast cancer (ICD-10 C50), colorectal cancer (ICD-10 C18-21), and prostate cancer (ICD-10 C61), which are the three commonest cancers in Qatar (2014) [29]. Cause-specific age-specific deaths for injuries were not available before 2000. As such, ASMRs between 2000–2014 for injuries, transport accidents (V1-V99), and falls (W0-W19) were estimated.

Estimated ASMRs and their standard errors were imported into the United States Surveillance, Epidemiology, and End Results Joinpoint Trend Analysis Software (version 4.4.0.0) [30]. Using Joinpoint regression, we assessed ASMR time trends by estimating annual percent change (APC) and average annual percent change (AAPC) in ASMR by period, nationality, and sex. Joinpoint software cannot process records with dependent variables (death rates) equal to zero [30]. As such, the years with mortality rates equal to zero were removed from the trends. When a trend was not significantly increasing or decreasing, it was considered constant. The significance of mortality trend changes were tested using Monte Carlo Permutation method (probability (p)-value threshold = 0.05). We provided contextual information that could explain all-cause mortality trends in Qataris and non-Qataris by reporting pertinent dates that marked Qatar's socioeconomic and healthcare system development [31, 32].

In order to identify significant differences in all-cause mortality between Qataris and non-Qataris, we estimated Comparative Mortality Figures (CMFs), which correspond to the ratio of ASMR in Qataris divided by the ASMR in non-Qataris [27]. We also computed 95% confidence intervals (CI) of CMFs and considered the ratio between ASMRs in Qataris and non-Qataris to be significant if the unity was not included within the 95% CI limits. Additionally, years of life lost (YLL), which is a summary measure of premature death were estimated by sex, age, and nationality due to all causes of death, neoplasms, cardiovascular and circulatory disease, and injuries in 2014 and 2015. YLL was estimated using WHO standard life expectancy at age of death, which provides life expectancy in years (L) in each age group of a standard population [33]. YLL (YLL = Number of deaths x L), which gives a higher weight to death at younger ages, brings the attention to those causes of death that are more common in younger age groups.

Results

During the study period, 26,673 deaths in over-20-year-old adults were recorded (15244 in non-Qataris and 11,429 in Qataris). Deaths in males were 2.7 times more frequent than in females (19,367 and 7,306 deaths). The annual average number of all-cause deaths was 1,159 deaths ranging from 632 (1989) to -2,070 (2014). In non-Qataris, annual total number of deaths increased about four-times, from 296 deaths (1989) to 1,438 deaths (2015). In Qataris, annual total number of deaths also increased but at a lower magnitude as compared to non-Qataris (from 336 deaths in1989 to 560 in 2015). In 2014, in Qatar's total population, injuries were responsible for 19.7% of deaths (92.6% in males), followed by cardiovascular and circulatory disease 17.8% (69.9% in males), and neoplasms 15.8% (46% in females).

In 2015, we estimated 60,716 YLL (82% in males) in Qatar's total population (S1 Table). In 2014, we estimated a similar total number and sex-distribution of YLL (S2 Table). During 2014, 29.2% of YLL were attributed to injuries, followed by cardiovascular and circulatory disease (14.4%), and neoplasms (11.8%). The first cause of premature mortality in Qatari and non-Qatari males was injury counting for 25.1% (2,043 YLL) and 34.9% (14,686 YLL) of the YLL due to all causes (8,132 YLL and 42,144 YLL, respectively) in 2014. The same year, the first cause of premature mortality in Qatari and non-Qatari females was neoplasms counting for 33.5% (1,724 YLL) and 25.1% (1,636 YLL) of the YLL due to all causes (5,140 YLL and 6,520 YLL, respectively).

Between 1989–2015, all-cause ASMR (per 100,000) significantly decreased in non-Qataris (from 346 to 229 in females and 402 to 133 in males); and in Qataris (from 2,754 to 382 in females and 4,086 to 506 in males; Fig 1a and 1b, Table 1). For 1989–2015, AAPC was -3.3% in non-Qataris and -7.7% in Qataris (both sexes), which led to an AAPC at -5.5% in Qatar's total population (p-values$<$0.05). Even if ASMR decreased two-times faster in Qataris than in non-Qataris, all-cause ASMRs in Qataris were always significantly higher than in non-Qataris (both sexes, $CIF_{Q/NQ}$ ranging from 7.7 in 1989 to 3.1 in 2015). However, the magnitude of the ratio decreased over time (both sexes). Thus, all-cause ASMR in Qatar's total population was always lower than in Qataris because of the mortality rates in non-Qataris, which includes mainly migrant workers. Overall, healthy worker effect seems to influence ASMR trends in Qatar's total population.

In Qatari males and females, all-cause ASMR trends have been steadily decreasing since 1989 (APC = -7.9% and -7.6%, p-values$<$0.05). In non-Qatari females, the decreasing trend was steady since 1989 but at a slower pace (APC = -2.2%, p-values$<$0.05). This decrease in mortality appears to follow Qatar's gradual healthcare system and socioeconomic development. Remarkably, this steady decrease in mortality in non-Qatari females occurred while

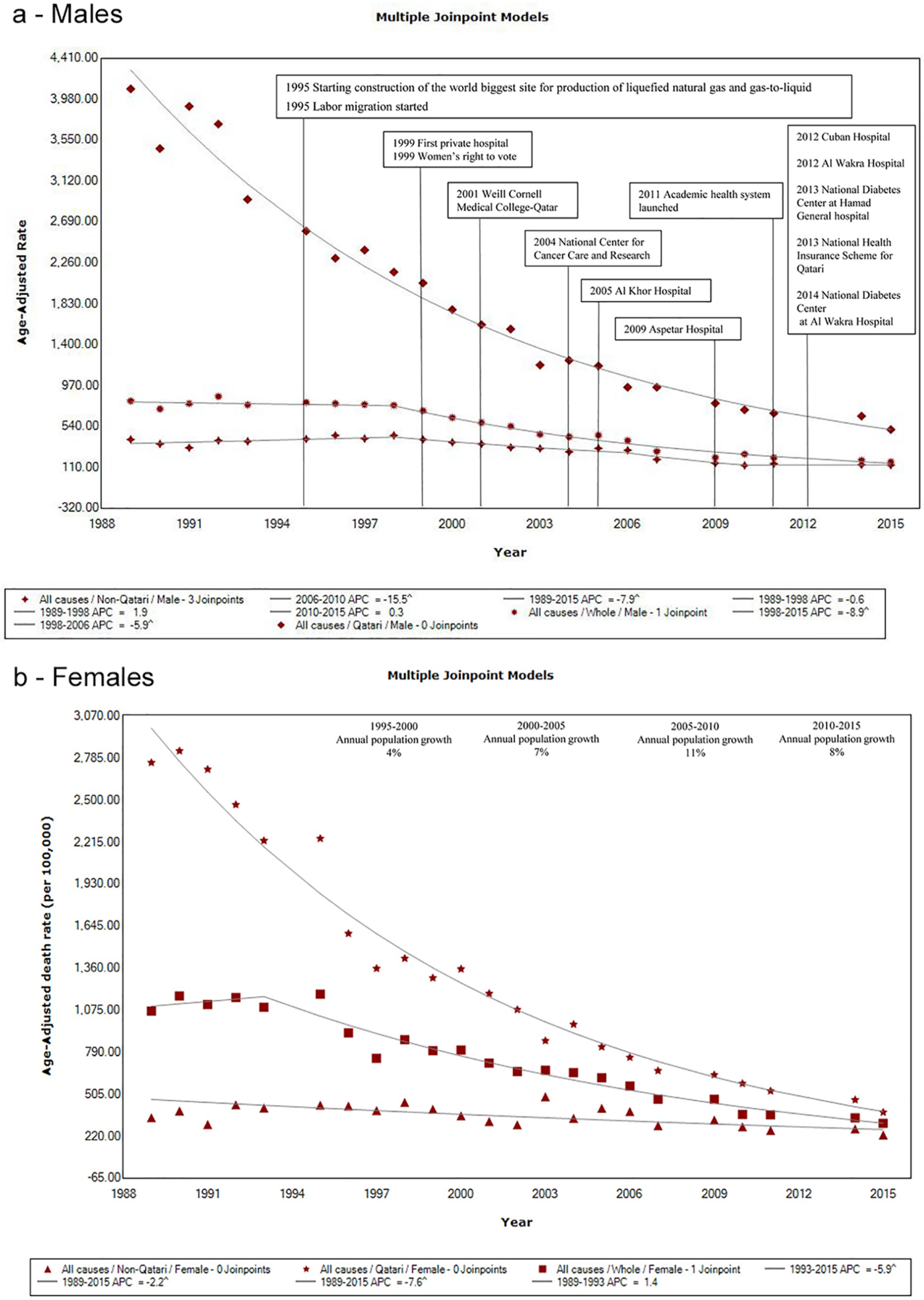

Fig 1. Trends in all-cause age-standardized moratlity rates (per 100,000) in males and females, 1989–2015 (Legend: ^: *p*-value<0.05). a- males. b- females. Data sources: Qatar Vital Statistics Annual Bulletins of Ministry of Development Planning and Statistics (MDPS) (http://www.mdps.gov.qa/en/statistics1/pages/topicslisting.aspx?parent=Population&child=BirthsDeaths)[22], MDPS's Census, Population, Housing, and Establishments annual reports (http://www.qix.gov.qa/portal/page/portal/QIXPOC/Documents/QIX%20Knowledge%20Base/Publication/Labor%20Force%20Researches/labor%20force%20sample%20survey)[25],

and the World Population Prospect 2015 Revision by United Nations (UN) Population Division (https://esa.un.org/unpd/wpp/Download/Standard/Population/) [4] Contextual information sources: Qatar Ministry of Public Health [31] and a review by Goodman and *al* [32].

https://doi.org/10.1371/journal.pone.0203996.g001

population growth in this population did not fluctuate in the same magnitude as in non-Qatari males. In non-Qatari males, all-cause ASMR trend decreased significantly between 1998–2010 (APC = -4.6, p-values<0.05) and reached a plateau thereafter. This significant decrease in mortality appears to have occurred after massive labor migration started. Between 2005–2010, population growth reached a peak at 28% in males. Interestingly, within the same period the decrease in mortality in non-Qatari males was the highest (AAPC = -13.7%, p-value<0.05). When population growth in males was below 5% in 1990–1995, 1995–2000, and 2010–2015, AAPCs for the same periods were not significant. The large increase in non-Qatari population within a short span of time could partially explain decreased ASMRs. Of note, ASMR is a weighted average of age-specific rates [27]. So, if the denominators (population size) within age groups were increasing dramatically during a short period of time due to the influx of young and/or healthy migrants while the numerators remained minimally affected, age-specific rates would also decrease. Healthy worker effect seems to influence ASMR trends in non-Qataris.

Between 1991–2014, cause-specific ASMR for cardiovascular and circulatory disease was higher in Qataris than in non-Qataris (both sexes), which resulted in higher ASMRs in Qataris compared to ASMRs in Qatar's total population (Tables 2 and 3). During this period, mortality trend declined significantly in non-Qatari females and in Qataris females

Table 1. Average annual percent change (AAPC) of all-cause age-standardized mortality trends in males.

		Males		Females	
Range	Nationality	AAPC (%)	Population growth (%)[1]	AAPC (%)	Population growth (%)[1]
1989–1995	Qatari	-7.9^	7.2	-7.6^	6.3
	Non-Qatari	1.9	0.7[2]	-2.2^	1.7[2]
	Total	-0.6	0.7[2]	-1.1	1.7[2]
1995–2000	Qatari	-7.9^	7.2	-7.6^	6.3
	Non-Qatari	-1.3	-	-2.2^	-
	Total	-2.1^	3.4	-5.9^	4.3
2000–2005	Qatari	-7.9^	7.2	-7.6^	6.3
	Non-Qatari	-5.9	8.9	-2.2^	6.9
	Total	-11.2^	8.9	-5.9^	6.9
2005–2010	Qatari	-7.9^	7.2	-7.6^	6.3
	Non-Qatari	-13.7^	27.7	-2.2^	11.2
	Total	-8.9^	27.7	-5.9^	11.2
2010–2015	Qatari	-7.9^	7.2	-7.6^	6.3
	Non-Qatari	0.2	4.4	-2.2^	8.1
	Total	-5.7^	4.4	-5.9^	8.1

Abbreviation: AAPC: Average annual percent change;

^: AAPC were statistically significant (p-value<0.05).

[1]: We assumed that non-Qatari population growth was similar to Qatar's population (total) growth,[4] as this population constituted the vast majority of Qatar's total population (73% in 1986[26] to 90% in 2016[5]). For Qatari population, we assumed constant increase in population between 1989–2015 as observed between 2006–2014.[25]

[2]: Population growth for the period 1990–1995.

https://doi.org/10.1371/journal.pone.0203996.t001

Table 2. Annual percent change in time trends of cause-specific age-standardized moratlity rates in Qatari and non-Qatari males, 1990–2014.

		Lower endpoint			Upper endpoint			
**Cause of death**	**Nationality**	**Year**	**Observed ASMR**	**Modeled ASMR**	**Year**	**Observed ASMR**	**Modeled ASMR**	**APC (%)**
**Cardiovascular and circulatory disease**	Qatari	1991	1780.5	2111.9	2014	220.0	99.0	-12.5^
	Non-Qatari	1991	149.6	159.6	1998	232.1	244.3	6.3
		1998	232.1	243.3	2009	25.2	23.9	-19.0^
		2009	25.2	23.9	2014	26.9	26.9	2.4
	Total	1991	360.4	384.2	1998	379.3	378.4	-0.2
		1998	379.3	378.4	2009	39.4	43.2	-17.9^
		2009	39.4	43.2	2014	35.6	35.7	-3.7
Cerebrovascular disease	Qatari	1991	412.3	478.7	2001	128.8	153.3	-10.8^
		2001	128.8	153.3	2005	17.2	11.1	-48.1
		2005	17.2	11.1	2009	30.3	30.4	28.6
		2009	30.3	30.4	2014	3.8	3.7	-34.5
	Non-Qatari	1991	26.1	57.9	2014	2.7	2.5	-12.7^
	Total	1991	75.4	82.7	1999	72.3	70.0	-2.1
		1999	72.3	70.0	2006	4.9	4.5	32.5^
		2006	4.9	4.5	2010	10.1	8.5	17.4
		2010	10.1	8.5	2014	2.8	2.7	-24.9
Hypertensive disease	Qatari	1990	54.9	97.1	2004	119.7	129.2	2.1
		2004	119.7	129.2	2007	13.0	18.1	-48.0^
		2007	13.0	18.1	2014	51.2	51.0	15.9
	Non-Qatari	1990	1.7	10.0	2002	35	30.5	9.8^
		2002	35.0	30.5	2009	2.7	2.0	-32.1^
		2009	2.7	2.0	2014	6.5	6.5	26.1
	Total	1990	8.5	21.3	2002	57	53.5	8.0^
		2002	57	53.5	2007	5.0	6.2	-35.0^
		2007	5.0	6.2	2014	10.9	10.2	7.4
Ischemic heart disease	Qatari	1991	902.2	983.1	2014	47.4	48.2	-12.3^
	Non-Qatari	1991	87.0	84.4	1998	130.5	139.9	7.4
		1998	130.5	138.9	2001	31.8	33.3	-37.1
		2001	31.8	33.3	2014	11.5	10.8	-8.6^
	Total	1991	191.8	195.4	1996	236.7	197.9	0.3
		1996	236.7	197.9	2014	14.7	12.5	-14.2^
**Neoplasms**	Qatari	1991	590.6	560.5	2014	78.8	69.9	-8.7^
	Non-Qatari	1991	14.3	47.5	2014	14.2	13.3	-5.4^
	Total	1991	98.6	134.2	2014	19.5	20.0	-8.0^
Colorectal cancer	Qatari	2000	27.1	16.6	2014	12.3	8.8	-4.4
	Non-Qatari	2000	1.9	1.7	2014	2.5	1.8	0.7
	Total	2000	6.7	6.1	2009	1	1.6	-14.0^
		2009	1	1.6	2014	3.3	3.3	16.3
Prostate cancer	Qatari	2000	27.1	21.4	2014	7.1	6.4	-8.3^
	Non-Qatari	2003	1	1.3	2014	1.2	1.1	-2
	Total	2000	5.1	3.7	2014	1.7	1.6	-6.1^
**Injuries**	Qatari	2000	115.6	155.1	2014	67.7	73.8	-5.2^
	Non-Qatari	2000	41.0	44.1	2005	75.6	74.7	11.1
		2005	75.6	74.7	2010	22.1	23.7	-20.5^
		2010	22.1	23.7	2014	24.7	25.4	1.7
	Total	2000	56.5	57.9	2005	83.3	87.4	8.6
		2005	83.3	87.4	2009	33.9	34.2	-20.9
		2009	33.9	34.2	2014	27.9	27.9	-4.0

(*Continued*)

Table 2. (Continued)

Cause of death	Nationality	Lower endpoint			Upper endpoint			APC (%)
		Year	Observed ASMR	Modeled ASMR	Year	Observed ASMR	Modeled ASMR	
Transport accidents	Qatari	2000	101.4	133	2014	53.8	61.1	-5.4^
	Non-Qatari	2000	36.2	32.0	2005	48.4	5.8	7.4
		2005	48.4	5.8	2009	15.4	14.8	-24.6
		2009	15.4	14.8	2014	13.3	13.8	-1.3
	Total	2000	49.7	47.9	2005	55.9	56.3	3.3
		2005	55.9	56.3	2009	20.2	21.4	-21.5^
		2009	20.2	21.4	2014	16.1	16.7	-4.8
Falls	Qatari	2000	9.1	7.8	2009	1.2	4.8	-5.2
	Non-Qatari	2001	5.1	9.8	2014	2.6	2.1	-11.3^
	Total	2000	2	9.1	2014	2.5	2.2	10.4

Abbreviations: ASMR: Age-standardized moratality rates (per 100,000); APC: annual percent changes

^: APC were statistically significant (p-value<0.05).

https://doi.org/10.1371/journal.pone.0203996.t002

and males (APC = -6.5%; -10.5%; and -12.5%, respectively; p-values<0.05). However, mortality in non-Qataris males decreased significantly between 1998–2009 (APC = -19.0%, p-value<0.05).

For cerebrovascular disease, cause-specific ASMR was lower in non-Qataris than in Qataris until 2011 in females and males, which resulted in lower ASMRs in Qatar's total population compared to ASMRs in Qataris. Mortality in non-Qatari males decreased significantly between 1998–2014 (APC = -12.7%), while in Qataris it decreased significantly between 1991–2001 (APC = -10.8%). In females, mortality was higher in Qataris than in non-Qataris (11.4 and 8.4/100,000, respectively) in 2014. Mortality rates in females were higher than in males (3.8 and 2.7/100,000, respectively).

For ischemic heart disease, cause-specific ASMR was lower in non-Qataris than in Qataris (both sexes), which resulted in lower ASMRs in Qatar's total population compared to ASMRs in Qataris. ASMRs decreased significantly in non-Qatari males since 2001 (p-value<0.05), while in Qataris (both sexes) and in non-Qatari females, decreased ASMRs were observed a decade earlier (p-value<0.05).

For hypertensive disease, cause-specific ASMR was lower in non-Qataris than in Qataris (both sexes) between 1990–2014, which resulted in lower ASMRs for Qatar's total population compared to ASMRs in Qataris. Similar decreasing and significant trends were observed in Qatari females and males and in non-Qatari females (APC = -12.3%; -11.5%; and -7.3%, respectively; p-value<0.05). In non-Qatari males the trend was constant until 2001 and a significant decrease in mortality was observed thereafter (APC = -8.6%; p-value<0.05).

For neoplasms, cause-specific ASMR was lower in non-Qataris than in Qataris (both sexes), which resulted in lower ASMRs in Qatar's total population compared to ASMRs in Qataris. A significant decrease was observed in non-Qatari males and in Qataris females and males since 1991 (APC = -5.4%; -8.6%; and -6.7%. respectively, p-value<0.05). In non-Qataris females, ASMRs were constant.

For female breast cancer, cause-specific ASMR was lower in non-Qataris than in Qataris, which resulted in lower ASMRs in Qatar's total population compared to ASMRs in Qataris. As mortality trends were constant in non-Qataris and significantly declining in Qataris (APC = -5.6%; p-value<0.05), in 2014, ASMRs in non-Qataris and Qataris were similar at 22.5 and

Table 3. Annual percent change in time trends of cause-specific age-standardized moratlity rates in Qatari and non-Qatari females, 1990–2014.

		Lower endpoint			Upper endpoint			
Cause of death	Nationality	Year	Observed ASMR	Modeled ASMR	Year	Observed ASMR	Modeled ASMR	APC (%)
**Cardiovascular and circulatory disease**	Qatari	1991	1155.2	1227.4	2014	111.3	95.1	-10.5^
	Non-Qatari	1991	153.21	246.2	2014	55.8	52.0	-6.5^
	Total	1991	491.8	611.6	2014	79.3	70.4	-9.0^
Cerebrovascular disease	Qatari	1991	270.1	411.9	2014	11.4	10.1	-14.9^
	Non-Qatari	1991	50.2	77.9	2014	8.4	8.1	-9.4^
	Total	1991	124.7	162.1	1999	87.8	94.6	-6.5
		1999	87.8	94.6	2005	2.6	6.6	-35.8^
		2005	2.6	6.6	2009	19.7	18.6	29.6
		2009	19.7	18.6	2014	10.1	10.3	-11.6
Ischemic heart disease	Qatari	1991	616.4	537.0	2014	30.7	32.2	-11.5^
	Non-Qatari	1991	62.4	95.8	2014	9.8	16.6	-7.3^
	Total	1991	249.3	260.4	2014	17.8	22.8	-10.0^
Hypertensive disease	Qatari	1990	93.4	88.5	2004	135.0	152.2	3.9
		2004	135.0	152.2	2009	18.4	14.4	-37.6^
		2009	18.4	14.4		51.4	51.5	-29.0^
	Non-Qatari	1990	9.5	19.7	2003	66.5	67.9	10.0^
		2003	66.5	67.9	2009	4.9	5.9	-33.4
		2009	4.9	5.9	2014	22.0	22.4	30.4
	Total	1990	37.9	43.0	2003	99.1	113.0	7.7^
		2003	99.1	113.0	2009	9.8	10.3	-32.9^
		2009	9.8	10.3	2014	34.3	34.4	27.2
**Neoplasms**	Qatari	1991	257.1	349.6	2014	86.2	71.1	-6.7^
	Non-Qatari	1991	31.8	76.5	2014	69.8	70.2	-0.4
	Total	1991	107.9	188.3	2014	74.6	64.6	-4.5^
Breast Cancer	Qatari	2000	41.1	43.7	2014	23.7	19.4	-5.6^
	Non-Qatari	2000	20.0	12.1	2014	22.5	24.1	5.1
	Total	2000	28.1	24.0	2014	22.3	20	-1.3
Colorectal cancer	Qatari	2000	20.6	15.6	2014	5.2	12.2	-1.7
	Non-Qatari	2000	11.4	9.8	2014	10.8	9.0	-0.6
	Total	2000	15.6	11.4	2014	8.2	8.9	-1.7
**Injuries**	Qatari	2000	30.6	39.3	2014	14.3	12.2	-8.0^
	Non-Qatari	2000	3.1	18.3	2014	7.1	8.6	-5.3
	Total	2000	15	28.0	2014	8.7	8.7	-8.0^
Transport accidents	Qatari	2000	30.5	26.3	2014	11.3	9.9	-6.8^
	Non-Qatari	2001	15.4	13.7	2014	3.7	2.8	-11.6^
	Total	2000	13.3	18.4	2014	5.9	4.9	-9.0^
Falls	Qatari	2005	3.9	-	2005	3.9	-	0
	Non-Qatari	2007	0.5	1.6	2014	0.4	0.6	-11.8
	Total	2005	2	1.6	2014	0.3	0.5	-11.9

Abbreviations: ASMR: Age-standardized moratality rates (per 100,000); APC: annual percent changes

^: APC were statistically significant (p-value<0.05).

https://doi.org/10.1371/journal.pone.0203996.t003

23.7/100,000, respectively. These trends resulted in a constant mortality rate trend for Qatar's female population.

For prostate cancer, cause-specific ASMR was lower in non-Qataris than in Qataris, which resulted in lower ASMRs in Qatar's total population compared to ASMRs in Qataris.

Mortality in non-Qataris, of which the vast majority is constituted of young people, remained stable. However, in Qataris, mortality trend declined significantly since 2000 (APC = -8.3%; *p*-value<0.05).

For colorectal cancer, cause-specific ASMR was lower in non-Qataris than in Qataris, which resulted in lower ASMRs in Qatar's total population compared to ASMRs in Qataris. The exception to this trend was in the mid-2000s and in 2014, when ASMRs were lower in Qatari females than in non-Qatari females. Modeled trends were constant in Qatari and non-Qatari males and females.

For injuries, cause-specific ASMR was lower in non-Qataris than in Qataris (both sexes), which resulted in lower ASMRs in Qatar's total population compared to ASMRs in Qataris. Qatari males had the highest ASMRs during the study period comparing to Qatari-females and non-Qatari males and females. Mortality decreased significantly in Qatari females and males since 2000 (APC = -8.0% and -5.2%, respectively; *p*-value<0.05). Between 2000–2014, in non-Qatari females and males, mortality trends were constant; except for males between 2005–2010 (APC = -20.5%, *p*-value<0.05).

For all falls, cause-specific ASMR in males was higher in non-Qataris than in Qataris, which resulted in higher ASMRs in Qatar's total population compared to ASMRs in Qataris. Deaths from falls were recorded for Qatari females only in 2005 (ASMR = 3.9/100,000). In non-Qatari females, deaths were recorded between 2007 and 2014; and ASMRs (per 100,000) ranged from 0.4 in 2014 and 2.5 in 2010 (modeled trend was constant). In Qatari males, deaths from falls have not been recorded since 2009 (ASMR = 1.2/100,000). In non-Qataris, mortality decreased significantly in males between 2001–2014 from 5.1 to 2.6/100,000 (APC = -11.3%, *p*-value<0.05).

For transport injuries, cause-specific ASMR was lower in non-Qataris than in Qataris (both sexes), which resulted in lower ASMRs in Qatar's total population compared to ASMRs in Qataris. Mortality trends decreased significantly in non-Qatari females and in Qatari females and males (APC = -11.6%; -6.8%; and -5.4%, respectively; *p*-value<0.05), while it was constant in non-Qatari males. In 2014, in males, ASMR was 13.4 and 53.8/100,000 in non-Qataris and Qataris, respectively. In females, it was 3.7 and 11.3/100,000 respectively.

Discussion

Our analysis of Qatar's vital registration data provides valuable insight on mortality trends in Qataris and non-Qataris (migrants). As reported elsewhere [10, 11], number of deaths did increase in non-Qataris but this is true also for Qataris. These increased numbers were higher in magnitude for non-Qataris. However, all-cause ASMRs in non-Qatari females like in Qataris (both sexes) has steadily decreased over the last 27 years, probably owing to socioeconomic healthcare system development. In non-Qatari males, rapid demographic changes probably influenced the reduction in ASMRs because of the healthy migrant effect especially during the migration peak in 2005–2010 [34]. From 2010, cause-specific ASMRs significantly decreased for neoplasms.

The healthy migrant effect has been described in other countries such as Australia [35], Finland [36], and Belgium [37] as a lower mortality risk in migrants comparing with people who were born in the study country. In the countries of the Gulf Cooperation Council, we demonstrated previously that country-level ASMR trends were associated with population size trends likely because of the healthy migrant effect [34]. Another study emphasized that long-term residents (more than ten years of residence) in the United Arab Emirates acculturate their lifestyle with the one in the host country, which lead to a decrease of the healthy migrant effect [38]. Remarkably, in Qatar, only 14% of the non-Qatari population was long-term resident (more

than ten years of residence) [39]. The percentage of short-term resident migrants (four years or less of residence) in Qatar was about 70% in 2010–20% higher than in the United Arab Emirates [39]. Here, we demonstrate that the reduction in Qatar's total population mortality (all causes, cardiovascular and circulatory disease, and neoplasms) could be partially attributed to the healthy migrant effect because the non-Qatari population demonstrates lower mortality rates for all causes, cardiovascular and circulatory disease, and neoplasms comparing to the Qatari population. The lower ASMRs in non-Qataris resulted in an even greater decrease in Qatar's total population ASMRs that could have been at higher levels otherwise. Several factors can explain the lower mortality observed in non-Qataris: migrant health screening by Qatar (selected newcomers to stay and work are negative for HIV, hepatitis B and C, syphilis, and tuberculosis); economic migrant self-selection (physically and mentally healthy individuals willing to migrate for work); and age profile (88% of the non-Qataris were aged 20–49 years in 2015) [25,35, 40, 41].

For injuries, mortality in non-Qatari males and females was lower than in Qatari males during the study period. In 2014, mortality due to falls was 0.4/100,000 in females and 2.6/100,000 in males. Remarkably, mortality due to falls in non-Qatari males decreased significantly suggesting safety improvement in the work environment. Non-Qatari males' work environment seems to have improved over time. In 2010–2013, yearly fatal occupational rate was 1.6/100,000 in the only designated hospital for major injury treatment in Qatar [42, 43]. This is half what is reported in the United States (3.4/100,000 in 2012) [44]. For transport accidents, mortality was lower in non-Qataris most probably reflecting the differential in the mode of transportation (collective transportation in non-Qatari workers versus individual vehicles in Qataris).

The influx of migrants has enabled Qatar to address the labor shortfall in the country. This migration emphasizes the substantial levels of economic growth observed in the country in the last decades [26]. Mortality among non-Qataris decreased, likely due to the healthy worker effect and the socioeconomic development of the country but also due to the implementation of laws to protect non-Qataris. Several Ministerial decisions were launched in the last decade to enhance the quality of life thus leading to improved health and safety of the workers in Qatar. For instance, since 2014, employers are required to provide adequate worker accommodations equipped with fire extinguishers, smoke detectors and fire alarm systems. Additionally, a health and safety officer must be appointed for each accommodation site which has 1200 workers residing on site [45]. These efforts from the government should continue along with regulatory checks in order to sustain the mortality decline in non-Qataris.

Our study demonstrated that while young adult males in Qatar die predominantly from injuries, young adult females die from neoplasms. These identified causes of death are for a large part preventable and should be addressed appropriately to lower premature mortality among young adults in Qatar. In 2013, a law was enacted to ensure the implementation of a mandatory health insurance system of basic health services to all residents of Qatar [46]. As treatment for chronic diseases is free for non-Qataris, preventing premature mortality among non-Qatari females should focus on screening for neoplasms. Scaling-up of screening is likely to increase the number of diagnosed cancer recognized at an earlier stage that can potentially be cured.

Regarding Qatari citizens, the access to free healthcare, housing grants, and subsidized education that accompanied the socioeconomic development of the country [26] has likely contributed to mortality decline. Concerning Qatari females' premature mortality, scaling up screening may be a relevant strategy to identify cancer cases at an earlier stage, as such treatment can be initiated early and the cancer cured potentially. In Qataris males, transport accidents appear to be the major killer. Lack of compliance pertaining to seat belt use and the use

of mobile phones amongst Qatar's drivers was reported [47]. Hence, we recommend the implementation of more aggressive and consistent national awareness campaigns pertaining to road safety and appropriate enforcement of laws related to road safety.

The strength of our study is that we emphasize demographic specificities in Qatar that should be taken into consideration when developing, implementing, and monitoring public health programs. Policy makers may use the estimated mortality rates and be able to distinguish between nationals (Qataris) and migrants (non-Qataris) when developing strategies to address premature mortality causes. Our analysis is relatively exhaustive unlike previous reports like GBD 2015 [48], which does not provide mortality estimates by sub-populations (Qataris versus non-Qataris). As such, we were able to compare mortality in the total population with mortality among Qatari and non-Qatari populations to further demonstrate our hypothesis of the healthy migrant effect [34].

The use of these secondary mortality data has several limitations such as missing data for several years and causes of death. Data validity is also difficult to assess because all data are retrieved from the same source (MDPS) [26]. Nevertheless, according to the UN Statistics Division, the coverage rate of the civil registration of births and deaths had reached 97% since the 1990s [26, 49]. Additionally, ASMRs in non-Qataris might be underestimated: non-Qataris come to Qatar for work; so, unhealthy individuals return to their country of origin when they are not able to work productively. Furthermore, individuals reaching the retirement age return to their country of origin. Misclassified cause of death was probably also reported in death certificates [50] introducing bias in vital registration data. However, these data limitations would affect both Qataris and non-Qataris. Nevertheless, the advantage of using such unique data in our analysis is to assess mortality trends at population-level. Hence, the strength of our work is the comparison of mortality trends among Qataris and non-Qataris over an extensive period.

Qatar's overall mortality trends appear to be partially attributed to the healthy migrant effect, as migrants have lower mortality rates. Nevertheless, Qatar's socioeconomic development does also appear to drive mortality decline in both Qataris (nationals) and non-Qataris (migrants). Yet, premature mortality remains and might be addressed by scaling-up screening programs and encouraging healthy lifestyle and road safety.

Supporting information

S1 Fig. Qatar's population structure by age group, sex, and nationality in 2014. Data source: MDPS's Census, Population, Housing, and Establishments annual report for the year 2014 [25]. (TIF)

S1 Table. Number of deaths and years of life lost due to all causes by sex and age group in Qataris and non-Qataris, in 2014 and 2015.
(XLSX)

S2 Table. Number of deaths and years of life lost due to neoplasms, cardiovascular and circulatory diseases, and injuries, by sex and age group in Qataris and non-Qataris, 2014.
(XLSX)

Author Contributions

Conceptualization: Karima Chaabna, Sohaila Cheema, Ravinder Mamtani.

Data curation: Karima Chaabna, Amit Abraham, Hekmat Alrouh.

Formal analysis: Karima Chaabna.

Methodology: Karima Chaabna.

Software: Karima Chaabna.

Supervision: Sohaila Cheema, Ravinder Mamtani.

Validation: Karima Chaabna, Sohaila Cheema, Ravinder Mamtani.

Writing – original draft: Karima Chaabna.

Writing – review & editing: Karima Chaabna, Sohaila Cheema, Amit Abraham, Hekmat Alrouh, Ravinder Mamtani.

References

1. Salam AA, Elsegaey I, Khraif R, AlMutairi A, Aldosari A. Components and public health impact of population growth in the Arab world. PloS One. 2015; 10(5):e0124944. https://doi.org/10.1371/journal.pone.0124944 PMID: 25993053.
2. Migration Policy Institute tabulation of data from the United Nations. Trends in International Migrant Stock: Migrants by Destination and Origin (United Nations database, POP/DB/MIG/Stock/Rev.2015): Department of Economic and Social Affairs,. 2015. http://www.migrationpolicy.org/programs/data-hub/charts/top-25-destinations-international-migrants.
3. Walker L. Ministry: Qatar's population increased by 40 percent since 2010 Doha, Qatar: Doha News; 2015. https://dohanews.co/tag/statistics/.
4. United Nations. Total Population—Both Sexes. De facto population in a country, area or region as of 1 July of the year indicated. Figures are presented in thousands. World Population Prospects: The 2015 Revision 2016. https://esa.un.org/unpd/wpp/Download/Standard/Population/.
5. Ministry of Development Planning and Statistics. Sample Labor Force Survey 2014 Qatar2014 [updated February 15, 2017]. http://www.qix.gov.qa/portal/page/portal/QIXPOC/Documents/QIX%20Knowledge%20Base/Publication/Labor%20Force%20Researches/labor%20force%20sample%20survey/Source_QSA/Labour_Force_Statistics_MDPS_Bu_AE_2014.pdf.
6. De Bel-Air F. Gulf Labour Markets and Migration (GLMM). Demography, Migration, and the Labour Market in the UAE. GLMM—EN—No. 7/2015. San Domenico di Fiesole, Italy: European University Institute and Gulf Research Center (GRC), 2015.
7. Adams RH. The determinants of international remittances in developping countries. World Development. 2009; 37(1):93–103.
8. Naufal GS. Chapter 27—The Economics of Migration in the Gulf Cooperation Council Countries. Handbook of the Economics of International Migration. 2015; 1:1597–640. https://doi.org/10.1016/B978-0-444-53768-3.00027-8.
9. Priya DSouza Consultancy. Population of Qatar by nationality—2017 report. Doha, Qatar: 2017.
10. International Trades Union Confederation. The case against Qatar. Host of the FIFA 2022 World Cup. 2014.
11. DLA Piper. Migrant Labour in the Construction Sector in the State of Qatar. Doha, Qatar: 2014.
12. The World Bank. Diabetes prevalence (% of population ages 20 to 79) 2016 [cited 2017 January, 11 2017]. http://data.worldbank.org/indicator/SH.STA.DIAB.ZS.
13. Supreme Council of Health. WHO Qatar STEPWISE Report 2012 chronic disease risk factor surveillance Doha, Qatar: 2013.
14. World Health Organisation. Obesity and overweight fact sheet Geneva, Switzerland2016. http://www.who.int/mediacentre/factsheets/fs311/en/.
15. Ministry of Health. WHO STEPWISE Approach to chronic disease risk factor surveillance. Country-specific standard report. Kuwait. Kuwait: 2015.
16. Ministry of Health. WHO STEPWISE Approach to chronic disease risk factor surveillance. Country-specific standard report. Bahrain. Bahrain: 2007.
17. World Health Organisation. Noncommunicable Diseases (NCD) Country Profiles, 2014 Switzerland2014 [cited 2017]. http://www.who.int/nmh/countries/are_en.pdf.
18. Ministry of Health. WHO STEPWISE Approach to chronic disease risk factor surveillance. Country-specific standard report. Bahrain. Oman: 2006.
19. Ministry of Health. WHO STEPWISE Approach to chronic disease risk factor surveillance. Country-specific standard report. Saudi Arabia. Saudi Arabia: 2005.

20. Rahman S, Salameh K, Bener A, El Ansari W. Socioeconomic associations of improved maternal, neonatal, and perinatal survival in Qatar. Int J Womens Health. 2010; 2:311–8. https://doi.org/10.2147/IJWH.S12426 PMID: 21151678.

21. Institute for Health Metrics and Evaluation GBD Compare | Viz Hub: University of Washington; 2017. http://vizhub.healthdata.org/gbd-compare/.

22. Ministry of Development Planning and Statistics. Vital Statistics Annual Bulletin (Births and Deaths) 1989–2015 Qatar1989-2015 [updated January 22, 2017]. http://www.mdps.gov.qa/en/statistics1/pages/topicslisting.aspx?parent=Population&child=BirthsDeaths.

23. World Health Organisation. International Statistical Classification of Diseases and Related Health Problems. 9th Revision. Geneva, Switzerland2015.

24. World Health Organisation. International Statistical Classification of Diseases and Related Health Problems. 10th Revision. Geneva, Switzerland2016.

25. Bureau of Statistics. Sample Labor Force Surveys Qatar2006-2015 [updated February 15, 2017]. http://www.qix.gov.qa/portal/page/portal/QIXPOC/Documents/QIX%20Knowledge%20Base/Publication/Labor%20Force%20Researches/labor%20force%20sample%20survey.

26. De Bel-Air F. Gulf Labour Markets and Migration (GLMM). Demography, migration and labour market in Qatar. GLMM—EN—No. 8/2014. San Domenico di Fiesole, Italy: European University Institute and Gulf Research Center (GRC), 2015.

27. Boniol MH, H. Age-standardization and denominator. In: International Agency for Research on Cancer VI, editor. Cancer incidence in five continents. IARC Scientific Publication. 160. Lyon, Frane: International Agency for Research on Cancer; 2007. p. 99–101.

28. Dos Santos Silva I. Conventional standard populations used for international comparisons. Cancer Epidemiology: Principles and Methods. Lyon, France: International Agency for Research on Cancer. World Health Organization; 1999. p. 82.

29. National Cancer Program. Qatar National Cancer Registry. Cancer Incidence 2014. Doha, Qatar: 2016.

30. National Cancer Institute. Joinpoint Trend Analysis Software 2017 [cited 2017 March 15, 2017]. https://surveillance.cancer.gov/joinpoint/.

31. Qatar Ministry of Public Health. Qatar Helath Sytem. Key organizations Doha, Qatar2017 [cited 2017 April 24, 2017]. https://www.moph.gov.qa/health-services/services-to-public/qatar-health-system.

32. Goodman A. The development of the Qatar Healthcare System: A review of the literature. International Journal of Clinical Medicine. 2015; 6:177–85. http://dx.doi.org/10.4236/ijcm.2015.63023.

33. World Health Organisation. Global Health Observatory data repository. Life tables by country: World Health Organization; 2016 [updated June 15, 2016April 24, 2017]. http://apps.who.int/gho/data/view.main.61180?lang=en.

34. Chaabna K, Cheema S, Mamtani R. Migrants, healthy worker effect, and mortality trends in the Gulf Cooperation Council countries. PLoS One. 2017; 12(6):e0179711. https://doi.org/10.1371/journal.pone.0179711 PMID: 28632794.

35. Page A, Begg S, Taylor R, Lopez AD. Global comparative assessments of life expectancy: the impact of migration with reference to Australia. Bull World Health Organ. 2007; 85(6):474–81. https://doi.org/10.2471/BLT.06.036202 PMID: 17639245.

36. Lehti V, Gissler M, Markkula N, Suvisaari J. Mortality and causes of death among the migrant population of Finland in 2011–13. Eur J Public Health. 2017; 27(1):117–23. https://doi.org/10.1093/eurpub/ckw196 PMID: 28177440.

37. Reus-Pons M, Vandenheede H, Janssen F, Kibele EU. Differences in mortality between groups of older migrants and older non-migrants in Belgium, 2001–09. Eur J Public Health. 2016; 26(6):992–1000. https://doi.org/10.1093/eurpub/ckw076 PMID: 27312258.

38. Shah SM, Loney T, Dhaheri SA, Vatanparast H, Elbarazi I, Agarwal M, et al. Association between acculturation, obesity and cardiovascular risk factors among male South Asian migrants in the United Arab Emirates—a cross-sectional study. BMC Public Health. 2015; 15:204. Epub 2015/04/18. https://doi.org/10.1186/s12889-015-1568-x PMID: 25885030.

39. Gulf Labour Markets and Migration. Demographic and Economic Database 2013 [cited 2018 17 May 2018]. http://gulfmigration.eu/category/database_demoeco/.

40. Mininstry of Public Health. Medical commission Doha, Qatar2017 [cited 2017 January, 11 2017]. https://www.moph.gov.qa/health-services/services-to-public/medical-commission/medical-commission.

41. Cheswick BR. Are Immigrants Favorably Self-Selected? The American Economic Review. 1999; 89 (9):181–5.

42. Al-Thani H, El-Menyar A, Consunji R, Mekkodathil A, Peralta R, Allen KA, et al. Epidemiology of occupational injuries by nationality in Qatar: Evidence for focused occupational safety programmes. Injury. 2015; 46(9):1806–13. Epub 2015/05/07. https://doi.org/10.1016/j.injury.2015.04.023 PMID: 25943291.
43. Mamtani R, Alrouh H, Cheema S, Lowenfels AB. Migrant mortality in Qatar. Injury. 2015; 46(12):2521–2. Epub 2015/11/02. https://doi.org/10.1016/j.injury.2015.10.017 PMID: 26520361.
44. National census of fatal occupational injuries in 2013. News release. United States: U.S. Department of Labor, Bureau of Labor Statistics, 2014 11 September 2014. Report No.: USDL-14-1674.
45. Martorella M. Qatar: Ministerial Decision No. 18 of 2014 Setting the Conditions and Specifications for Workers' Accommodations: Gulf Labour Markets and Migration; 2016 [8 August 2018]. http://gulfmigration.eu/qatar-ministerial-decision-no-18-of-2014setting-the-conditions-and-specifications-for-workers-accommodations/.
46. Martorella M. Qatar: Law No. 7 of 2013 on the Social Health Insurance System: Gulf Labour Markets and Migration; 2016 [8 August 2018]. http://gulfmigration.eu/qatar-law-no-7-of-2013on-the-social-health-insurance-system/.
47. Mahfoud ZR, Cheema S, Alrouh H, Al-Thani MH, Al-Thani AA, Mamtani R. Seat belt and mobile phone use among vehicle drivers in the city of Doha, Qatar: an observational study. BMC Public Health. 2015; 15:937. Epub 2015/09/24. https://doi.org/10.1186/s12889-015-2283-3 PMID: 26392362.
48. GBD 2015 Mortality and Causes of Death Collaborators. Global, regional, and national life expectancy, all-cause mortality, and cause-specific mortality for 249 causes of death, 1980–2015: a systematic analysis for the Global Burden of Disease Study 2015. Lancet. 2016; 388(10053):1459–544. https://doi.org/10.1016/S0140-6736(16)31012-1 PMID: 27733281.
49. Division US. Demographic and Social Statistics. Civil Registration and Vital Statistics: UN; 2016 [15 August 2018]. https://unstats.un.org/unsd/demographic-social/crvs/.
50. Wexelman BA, Eden E, Rose KM. Survey of New York City resident physicians on cause-of-death reporting, 2010. Prev Chronic Dis. 2013; 10:E76. https://doi.org/10.5888/pcd10.120288 PMID: 23660118.
